# Can BKPyV Infection Affect Neoplasm Transformation Among Kidney Transplant Recipients? A Case Series Study Report

**DOI:** 10.3390/jcm14238550

**Published:** 2025-12-02

**Authors:** Paweł Poznański, Maciej Wenta, Hanna Augustyniak-Bartosik, Dagna Rukasz, Agnieszka Hałoń, Katarzyna Kościelska-Kasprzak, Dorota Kamińska, Magdalena Krajewska

**Affiliations:** 1 Faculty of Medicine, Wroclaw University of Science and Technology, 50-370 Wrocław, Poland; 2 Department of Anesthesiology and Intensive Care, Pomeranian Hospitals, 84-200 Wejherowo, Poland; 3 Department of Clinical and Experimental Pathology, Wroclaw Medical University, 50-367 Wrocław, Poland

**Keywords:** BKPyV infection, polyomavirus-associated nephropathy, BKPyV-associated neoplasms, chromophobe renal cell carcinoma, kidney transplantation

## Abstract

**Background:** There is a great deal of knowledge regarding the development of polyomavirus-associated nephropathy and polyomavirus-associated hemorrhagic cystitis in transplant recipients with active BKPyV infection. However, recent studies have revealed a potential association between BKPyV reactivation and certain malignancies, including transitional cell carcinoma, malignant melanoma, colorectal cancer, and prostate cancer. This study aimed to identify a potential link between BKPyV infection and oncogenic transformation in kidney transplant recipients. **Methods:** Presentation of a case series of kidney transplant recipients diagnosed with polyomavirus-associated nephropathy who developed neoplasms after transplantation. **Results:** Positive immunohistochemical reactions confirmed the presence of polyomavirus large T antigen in tissue samples from all three patients’ cancers. Furthermore, a case of chromophobe renal cell carcinoma presenting BKPyV proteins in cancer cells was observed for the first time in the literature. **Conclusions:** BKPyV reactivation was found to be associated with the development of both urothelial cancer, which originates directly from the BKPyV-infected site, and colorectal cancer.

## 1. Introduction

BK poliomavirus (BKPyV), first described by Gardner et al. in 1971, was the first human polyomavirus (PyV) to be identified [1]. It was isolated from a kidney transplant recipient with ureteric stenosis and was named after this patient’s initials [1]. Poliomaviruses consist of a circular, double-stranded DNA genome comprising early and late transcription regions, preceded by a non-coding control region. The early region contains coding sequences for the large (PyLT-Ag) and small tumor antigens. BKPyV belongs to the Polyomaviridae family, alongside JCV, KIV, WUV, MCV and SV40. First contact with BKPyV usually occurs in childhood as asymptomatic seroconversion [2,3]. The seroprevalence of BKPyV reaches 90% among the general population [4]. Primary BKPyV infection can occur via the respiratory or oral route, as well as transplacentally and through blood transfusions [5,6,7]. Reactivation of BKPyV infection only occurs within immunocompromised hosts, particularly following transplantation, but also during pregnancy, HIV infection and diabetes mellitus. In the era of stronger immunosuppressive drugs, such as polyclonal induction agents, mycophenolate and tacrolimus, BKPyV reactivation may be facilitated [8,9].

Among kidney transplant recipients, reactivation of a primary BKPyV infection and/or transmission of a BKPyV strain of donor origin can lead to the development of BKPyV replication and BKPyV-related disease. BKPyV is a causative factor in polyomavirus-associated nephropathy (PyVAN) and ureteral stenosis in kidney transplant recipients, and polyomavirus-associated hemorrhagic cystitis in allogeneic hematopoietic stem cell recipients [2]. BKPyV has also been reported to cause retinitis, hemophagocytic syndrome, multiple organ failure, pneumonia, carcinogenesis, and progressive multifocal leukoencephalopathy (PML). However, most recent cases of PML have been attributed to JCV [2,10,11,12]

Kidney transplant recipients (KTRs) have a two- to threefold increased risk of malignancies compared to the general population. The cumulative incidence of any non-skin malignancy at month 36 after transplantation is 7.5%, with cancer rates similar to those of people 20–30 years older who have not undergone transplantation [13]. Recently, a growing number of publications have reported the possible oncogenic potential of BKPyV. In most cases, these tumors have been derived from the uroepithelium; however, malignant melanoma, colorectal and prostate cancers, brain tumors, pancreatic tumors, and Kaposi’s sarcoma have also been described [14,15,16,17].

We present herein a case series of kidney transplant recipients (KTRs) who were transplanted between 2002 and 2004 and who were later (between 2009 and 2013) suspected of having polyomavirus-associated nephropathy (PyVAN). These recipients developed BKPyV-associated neoplasms after transplantation, including the first reported case of a rare renal tumor: chromophobe renal cell carcinoma.

## 2. Methods

Patients suspected of having polyomavirus-associated nephropathy (PyVAN) were retrospectively identified from a single transplant center. The study included individuals who, between 2009 and 2013, developed a non-cutaneous neoplasm and were treated at the same institution, allowing access to archived tissue samples for additional staining.

The case descriptions were based on a retrospective review of anonymized clinical, laboratory, and histopathological data, conducted with approval from the local Ethics Committee. PyVAN suspicion was defined as a blood or urine viral load ≥10^6^ copies/mL (Polyomavirus BK Real Time-PCR Kit, Shanghai ZHIJIANG Biotechnology Co., Ltd., Shanghai, China) accompanied by an increase in serum creatinine of ≥50% or ≥1 mg/dL from baseline (which was according to the definition of suspicion at that time), and/or confirmed by histopathological examination.

Immunohistochemical staining for polyomavirus large T antigen (PyLT-Ag) was performed on paraffin-embedded neoplastic tissue samples obtained from recipients with PyVAN, using an anti-SV40 T antigen monoclonal antibody (DP02 Anti-SV40 T Antigen [Ab-2], Mouse mAb [PAb416]; Merck KGaA, Darmstadt, Germany).

## 3. Case Presentation

### 3.1. Patient Number 1

A 45-year-old male patient with end-stage renal disease due to MCGN underwent transplantation with 23 h of cold ischemic time from a 62-year-old deceased male donor. The immunological risk factors were four donor-recipient HLA mismatches and 4% panel reactive antibodies. The immunosuppression protocol comprised cyclosporine A, glucocorticoids, and azathioprine. Satisfactory graft function was achieved early on, with a serum creatinine concentration of 1.7–1.9 mg/dL. Three months after the kidney transplant, acute rejection occurred, with a serum creatinine concentration of 8.66 mg/dL. A kidney biopsy revealed acute rejection, which was treated with pulses of methylprednisolone, resulting in only partial restoration of transplanted kidney function. The serum creatinine concentration after the rejection episode was 2.8–3.0 mg/dL, even after treatment. The immunosuppression regimen was converted to cyclosporine A, glucocorticoids, and mycophenolate mofetil. BKPyV viral load was detected in the plasma, and PyVAN was suspected based on viral load and proven in histological findings seven years after transplantation, when the serum creatinine concentration was 6.62 mg/dL. Two months after the PyVAN diagnosis, a renal tumor was identified in an ultrasound examination of the native kidney. Following nephrectomy, a histological diagnosis of chromophobe renal cell carcinoma of the native kidney was made.

### 3.2. Patient Number 2

The patient was a 52-year-old male with end-stage renal disease due to chronic glomerulonephritis (without biopsy-proven type). The transplantation was performed with 35 h of cold ischemic time from a 46-year-old female deceased donor. The immunological risk factors were six donor-recipient HLA mismatches and 0% panel reactive antibodies. The immunosuppression protocol comprised tacrolimus, glucocorticoids, and mycophenolate mofetil. Good graft function was achieved early on, with a serum creatinine concentration of 1.32 mg/dL three months after transplantation. In the fifth post-transplant month, an acute rejection episode (AR) occurred with a serum creatinine concentration of 4.3 mg/dL. The AR was treated with pulses of methylprednisolone, which led to a satisfactory resolution of transplanted kidney function. The serum creatinine concentration 12 months after transplantation was 2.6 mg/dL. BKPyV viral load was detected in plasma and urine specimens, and pyelonephritis was diagnosed due to laboratory findings eight years after transplantation, with a serum creatinine concentration of 3.1 mg/dL. At the same time, a diagnosis of papillary urothelial carcinoma was made. The immunosuppression protocol was converted to cyclosporine A, glucocorticoids, and everolimus, and PyVAN features developed eight years after transplantation. Two years later, a diagnosis of rectal adenocarcinoma was made based on histopathological findings.

### 3.3. Patient Number 3

The patient was a 44-year-old male with end-stage renal disease due to distal tubular acidosis with nephrocalcinosis. The transplant was performed with 15 h of cold ischemic time from a 28-year-old female donor who had died. The immunosuppression protocol comprised sirolimus, cyclosporine A, and glucocorticoids. Good graft function was achieved early on. BKPyV viral load was detected in the patient’s plasma and urine six years after transplantation, at which point PyVAN was suspected. At the same time, a diagnosis of papillary uroepithelial carcinoma of the bladder was also made.

Table 1 presents detailed descriptions of the recipients’ characteristics.

**Figure 1:**
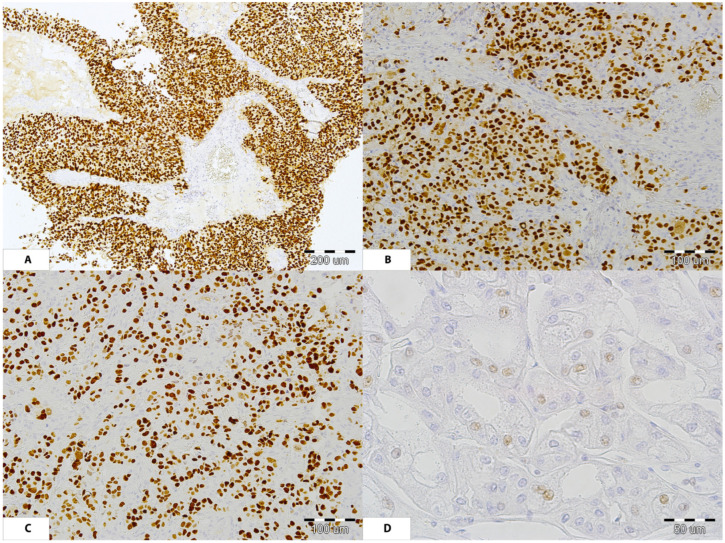
Presence of positive PyLT-Ag reaction in cancer tissues obtained from recipients with PyVAN. (**A**,**B**): Papillary urothelial carcinoma (patient No. 3) with complete strong nuclear PyLT-Ag expression within noninvasive papillary (**A**) and invasive solid (**B**) compartment of tumor. (**C**): Rectal adenocarcinoma (patient No. 2) invades submucosa with strong nuclear immunoreactivity within neoplastic glands. (**D**): Native kidney chromophobe renal cell carcinoma (patient No. 1) presenting weak nuclear reaction for PyLT-Ag in a few epithelial neoplastic cells.

To confirm the possible influence of BKPyV on cancer development, we tested archival tumor tissues for the presence of the polyomavirus large T-antigen (PyLT-Ag). We found a positive immunohistochemical reaction, confirming the presence of PyLT-Ag in all cancer tissue samples from all three patients (Figure 1).

## 4. Discussion

In total, 60–80% of the KTRs are BKPyV seropositive, even before transplantation, and 1–7% of them develop PyVAN. Recently the connection between BKPyV disease and carcinogenesis has been widely discussed [3,15,18,19].

Reactivation of BKPyV infection occurs only in immunocompromised hosts, especially after transplantation. Strong immunosuppressive drugs such as polyclonal induction immunoglobulins, mycophenolates, and tacrolimus may facilitate BKPyV reactivation [8]. Moreover, basing on in vitro trials, it is postulated that mTOR inhibitors, used in immunosuppressive regimes in solid organ transplantation recipients, can directly activate BKPyV replication in a dose-dependent manner. However, other studies indicate that the conversion to mTOR inhibitors can facilitate the BKPyV clearance and PyVAN treatment [20,21,22]. According to the Second International Consensus Guidelines on the Management of BK Polyomavirus in Kidney Transplantation there is insufficient data to evaluate the efficacy of switching to mTOR inhibitors for treating BKPyV-DNAemia or biopsy-proven BKPyV-nephropathy [23].

KTRs present 2–3-fold increased risk of malignancies compared to the general population. The cumulative incidence of any non-skin malignancy at month 36 after transplantation is 7.5% and cancer rates are similar to those among 20–30 years older non-transplanted people [24,25].

Some of the cancers follow viral infections. The molecular pathway of potential BKPyV-related oncogenesis was described. There are several hypothetical pathways affecting cell cycles with BKPyV-induced oncogenesis, including the “hit-and-run” mechanism, where BKPyV contributes to the onset of oncogenic transition and later is not detectable in tumor tissue, as well as the “passenger” hypothesis, according to which BKPyV facilitates progression of the already transformed cell. By interacting with tumor-suppressor proteins, such as p53 and pRb, PyLT-Ag can promote mechanisms that can contribute to tumor initiation and progression, i.e., uncontrolled proliferation and genomic instability [26,27,28]. Nonetheless, BKPyV can be detected in tumor cells as a “by-stander”, and it can be unrelated to the development of malignancy [29,30].

In our case series, we demonstrated that BKPyV replication can be involved in the development of urothelial cancer (which originates directly from the infected site) and colorectal cancer, despite there being no connection to the site of infection. Furthermore, we were the first to demonstrate that a rare variant of renal cancer, chromophobe cell carcinoma, is associated with the presence of BKPyV proteins in cancer cells.

Numerous single-case studies and case series have been published in the literature [31]. Emerson et al. described a case of collecting ducts of Bellini carcinoma (CDC) arising in association with PyVAN after kidney transplantation. They did not identify BKPyV DNA within neoplastic cells, but they observed BKPyV T-Ag protein expression in both intraepithelial and invasive neoplastic tissues [32]. The presence of BKPyV DNA in renal cell carcinomas of native as well as transplanted kidneys, including a metastatic tumor, was also reported [33]. BKPyV DNA positivity was significantly associated with the histological diagnosis of renal cell carcinomas, but not with that of bladder transitional cell carcinomas in a cohort of 90 patients with kidney or bladder cancer [34]. Moreover, BKPyV DNA has been detected in several other tumors in transplant recipients, e.g., colorectal tumors, lymphomas, pancreatic cancer, brain tumors, and a range of sarcomas [31].

Casini et al. described a series of 18 colorectal cancers and demonstrated that BKPyV DNA was present in 88.9% of cases, not only inside the tumor mass but also in the surrounding healthy tissues [9]. In our patients, we only found positive SV40 staining in the tumor cells and not in the surrounding tissue. In the case of rectal adenocarcinoma, we ruled out the possibility of tumor infiltration from the bladder to the rectum because none of the imaging or pathological methods revealed tumor spread from the bladder to the rectum. Furthermore, the patient was a transplant recipient with confirmed PyVAN. Therefore, the presence of BKPyV in this case links viral reactivation to cancer development.

It is also possible that BKPyV, PyVAN, and neoplasms develop independently as a result of intense immunosuppression. Most of the described BKPyV-related cancers occurred in recipients undergoing intense immunosuppression involving thymoglobulin during the induction phase, steroid pulses to treat acute rejection episodes, and long-term use of tacrolimus and mycophenolates. An analysis of over 164,000 solid organ transplant recipients found that the absolute cancer risk was slightly but significantly higher during the period from 2000 to 2008 than during the period from 1987 to 1999 [35]. Arguments against this theory were published following an analysis of tumor incidence in over 35,000 kidney transplant recipients (KTRs) in the USA. No difference in malignancy incidence was found for transplantations performed during 1999–2001 compared with 1995–1998. Furthermore, the type of initial immunosuppression did not increase the risk of non-skin malignancies [24].

However, we have found that another type of neoplasm that is possibly associated with active BKPyV replication—chromophobe cell carcinoma—which is a rare variant of renal carcinoma. The nuclear reaction between antibodies used to identify the virus (anti PyLT-Ag) and the antigen was weaker than in other cases but sill apparent in most cancer cells. It is possible that, due to the observed mutations in p53 in this type of cancer, a change occurred in this case resulting in a reduction in the interaction between SV40 and p53, which in turn led to a relative decrease in the number of SV40 antigen copies and a weaker immunohistochemical reaction [36].

Recently, several papers have analyzed the association between PyVs and carcinogenesis. While the association between PyVs and various types of cancer (e.g., breast, oral, and colorectal) was described, this does not indicate causation and requires further study in large prospective trials [37,38,39,40,41].

## 5. Limitations

The researchers do not have comparative data on the incidence of the aforementioned cancers from the center where the descriptions originated. Since BKPyV was only evaluated in cases of PyVAN susceptibility, reliable data on carcinogenesis incidence in BKPyV-positive and -negative KTRs is unavailable.

## 6. Conclusions

In our case series, we demonstrated that BKPyV reactivation can be involved in the development of urothelial cancer, which derives directly from the infected area, as well as colorectal cancer, ruling out a link with local infection. Furthermore, we were the first to demonstrate in the literature that a rare variant of renal cancer, chromophobe cell carcinoma, presents BKPyV proteins in cancer cells. In our patients, we found positive SV40 staining only in tumor cells and not in surrounding healthy tissue, which supports BKPyV-induced oncogenesis in these cases. To the best of our knowledge, this is the first report to describe the morphological features of chromophobe cell carcinoma that are positive for the SV40 PyV antigen BKPyV.

## Figures and Tables

**Table 1:** Characteristics of kidney transplant recipients who developed PyVAN and cancer.

	Patient No. 1	Patient No. 2	Patient No. 3
Cause of end-stage kidney disease	Membranoproliferative glomerulonephritis	Chronic glomerulonephritis	Distal tubular acidosis with nephrocalcinosis
Year of Tx	2003	2004	2002
Previous Tx	No	No	No
Recipient’s gender	Male	Male	Male
Donor’s gender	Male	Female	Female
Recipient’s age at Tx [y]	45	52	44
Donor’s age at Tx [y]	62	46	28
Cold ischemia time	23 h	35 h	15 h
No of episodes of AR previous to NPL	1	1	0
Time from Tx to AR [m]	3	5	-
IS protocol	CsA, AZA, GCs	TAC, MMF, GCs	Sirolimus, CsA, GCs
Time from Tx to NPL [y]	7	10	6
Age at the time of NPL	52	62	50
NPL type	Native kidney’s chromophobe cancer	Colorectal cancer	Urothelial cancer
IS conversion	CsA, MMF, GCs	Everolimus, CsA, GCs	CsA, MMF, GCs
Treatment of NPL	Surgery (nephrectomy)	Surgery (resection)	TURB, intravesical Bacillus Calmette–Guérin therapy
Return to dialysis	+	-	No data
Results	cured	cured	death during treatment

NPL—neoplasm; AR—acute rejection; IS—immunosuppression; Tx—transplantation; CsA—cyclosporine A; AZA—azathioprine; MMF—mycophenolate mofetil; TURB—transurethral resection of bladder tumor; GCs—glucocorticoids.

## Data Availability

The original contributions presented in the study are included in the article, further inquiries can be directed to the corresponding author.
